# 

**Published:** 1918-06-29

**Authors:** 


### CONTENTS.

PAGE

**Inter-Allied Scientific Food Commission** ... 273
**Independence or Contumacy?** ... ... ... 274
**Hospital and Institutional News** ... ... ... 275
Attempt to Commandeer a Hospital—A Hospital Treasurer's Proposal—Re-christening a London Hospital—The New Chairman of Chelsea Hospital for Women—The Proposed Hospital at the Cape—A Year of Immortality—Zeebrugge Heroes and the London Homœopathic—Pension Deduction for Sanatorium Treatment—Farm Colony for Combatants—An East-End Hospital Programme—The Influenza Epidemic—A Tuberculosis Policy for London—Gravel for the War Office—The Alleged Effect of State Subsidies in Australia—Suggested Paying Bed Scheme—The Cost of a District Nurse—Holidays and Cottage Hospitals—Nurses' Fees in Quarantine—A Massage School at Cape Town—This Week's Drug Market—To Our Readers.
**The Agitation for Unqualified Practitioners...** 279
Overdone Advocacy *v.* Common Sense.
**How Trench Fever is Conveyed** ... ... ... 281
The Louse: Its Habits and Methods.
**Medical Fees to Fellow-Practitioners** .. ... 282
Sir Henry Morris' Decision.
**The Crichton Royal Institution...** ... ... 283
Much Educational Matter Addressed to the Public.
**Nursing Affairs in Ireland** ... ... ... ... ... 284
Sir Arthur Stanley's Visit. Who Has Been "Done"?

PAGE

**The Matrons' and Sisters' Department** ... 285
District Nursing: III. The Labourer and Her Hire.
Round the Hospitals.
Air Service Secures Own Matron-in-Chief—State Registration Society's Proceedings—A Call to Yorkshire Nurses—The Trained Nurse as Housekeeper—New Order V.A.D. Hospitals.
**The South African Military Nursing Service** 289
**The Woman Worker's World** ... ... ... 289
Passing Topics and Movements.
**The Control of V.A.D. Hospitals** ... ... ... 290
**Orthopædics "Napoo" at Leicester** ... ... 291
**Hospital and Institutional Results** ... ... 291
**Legal Notes for Institutional Workers** ... 292
Panel Doctors and Insurance Committees.
**Parliament and the Profession** ... ... ... 292
**Gifts and Bequests** ... ... ... ... ... ... 293
**Passing Events and Topics** ... ... ... ... 293
**Matrons' and Sisters' Appointments** ... ... 294
**The College of Nursing (Limited by Guarantee)** 294
**Institutional Needs** ... ... ... ... ... ... 294
**Annual and Other Meetings** ... ... ... 294
**The Institutional Worker** ... ... (Suppl.) 1
The Organisation of a Crèche in a Munition Factory: Answer to May Question.

